# Disentangling stability and flexibility degrees in Parkinson’s disease using a computational postural control model

**DOI:** 10.1186/s12984-019-0574-0

**Published:** 2019-08-14

**Authors:** Zahra Rahmati, Alfred C. Schouten, Saeed Behzadipour, Ghorban Taghizadeh, Keikhosrow Firoozbakhsh

**Affiliations:** 1 Mechanical Engineering Department, Sharif University of Technology, Tehran, Iran; 2 Djawad Movafaghian Research Center in Rehab Technologies, Sharif University of Technology, Tehran, Iran; 3 Department of Biomechanical Engineering, Delft University of Technology, Delft, The Netherlands; 4 Department of Biomechanical Engineering, University of Twente, Enschede, The Netherlands; 5 School of Rehabilitation Sciences, Iran University of Medical Sciences, Tehran, Iran

**Keywords:** Parkinson’s disease, Postural control model, Posturography, Balance training, Stability and flexibility degrees, Power spectral density

## Abstract

**Background:**

Impaired postural control in Parkinson’s disease (PD) seriously compromises life quality. Although balance training improves mobility and postural stability, lack of quantitative studies on the neurophysiological mechanisms of balance training in PD impedes the development of patient-specific therapies. We evaluated the effects of a balance-training program using functional balance and mobility tests, posturography, and a postural control model.

**Methods:**

Center-of-pressure (COP) data of 40 PD patients before and after a 12-session balance-training program, and 20 healthy control subjects were recorded in four conditions with two tasks on a rigid surface (R-tasks) and two on foam. A postural control model was fitted to describe the posturography data. The model comprises a neuromuscular controller, a time delay, and a gain scaling the internal disturbance torque.

**Results:**

Patients’ axial rigidity before training resulted in slower COP velocity in R-tasks; which was reflected as lower internal torque gain. Furthermore, patients exhibited poor stability on foam, remarked by abnormal higher sway amplitude. Lower control parameters as well as higher time delay were responsible for patients’ abnormal high sway amplitude. Balance training improved all clinical scores on functional balance and mobility. Consistently, improved ‘flexibility’ appeared as enhanced sway velocity (increased internal torque gain). Balance training also helped patients to develop the ‘stability degree’ (increase control parameters), and to respond more quickly in unstable condition of stance on foam.

**Conclusions:**

Projection of the common posturography measures on a postural control model provided a quantitative framework for unraveling the neurophysiological factors and different recovery mechanisms in impaired postural control in PD.

**Electronic supplementary material:**

The online version of this article (10.1186/s12984-019-0574-0) contains supplementary material, which is available to authorized users.

## Introduction

Postural instability is regarded as the most detrimental symptom in Parkinson’s disease (PD) and hampers fundamental motor functions in daily activities [1]. Postural control is a multi-factor capability, with contribution from both balance control (body stabilization), and segmental orientation control (body orientation with respect to gravity). Diab et al. [2] reviewed the many contributing factors in the impaired postural control in PD. Convoluted emergence of these two components – orientation and stabilization –, along with multiple involving sub-systems, make the understanding of the underlying pathophysiology difficult; and asks for clear quantitative measures to disentangle the aspects of postural control [3, 4].

General treatments for PD such as pharmacotherapy and surgical brain stimulations have arguing drawbacks [5]. Notwithstanding that pharmacotherapy and surgery mitigate other PD symptoms such as tremor, rigidity, and bradykinesia, postural instability in PD is resistant to these two treatments [1, 2, 4, 5]. Even some studies indicates that postural instability is worsened by L-dopa therapy [6, 7]. Although it is well evidenced that balance training, can restore postural stability [5]; still a standardized program is under debate [4, 8]. Additionally, the multifaceted nature of postural control leads to different outcomes from different interventions, in which the influence of each balance exercise is not fully determined.

Clinical assessments of postural control, albeit simple and reliable, only observe physical performance; and lack the evaluation of neurophysiological causes of postural instability. Measures as posturography and gait analyses [9, 10] allow quantitative assessments of postural instability. However, static posturography has been mainly limited to the evaluation of medical/surgical treatments efficacy [11, 12]. Sway measures have less been attributed to clinical notions or at best remained in correlation-study level [1, 7, 11, 13, 14]. Posturography even ended in contradictory results [4], which further highlights their failure to link measures to the patient’s postural ‘stability degree’; that is to successfully address them to an applicable explanation of postural control in PD. This missing link can be found in other complex analyses of center-of-pressure (COP) data [15, 16].

Computational postural control models help us to precisely decode each facet of postural instability in a quantitative manner [3]; and to bind neurophysiological bases to quantitative biomarkers [17]. There have been few attempts to understand PD patients’ instability by postural control models [13, 18, 19]. Yet, none of these studies linked the model with clinical practices. The closest study in this regard considered elderly training [8] with focus on sensory integration in balance control. Computational study of postural instability during a training program provides objective tools for quantifying existing clinical understandings. Ultimately, predictive potency of models will pave the path for future design of optimal and patient-specific therapies.

This study aimed to investigate the neurophysiological aspects of the postural instability in PD, as well as how balance training can play a role in PD rehabilitation, with a quantitative approach. To this end, the effect of a balance-training program in PD was evaluated, using posturography and the postural control model of Maurer et al. [9]. The COP data of patients were collected before and after training, in addition to the same data from healthy control subjects (HCs); and each subject’s model parameters were identified. Both sway measures and postural control parameters were considered to provide a clinically-applicable implication for sway measures.

## Methods

The COP data from the patient group before and after a balance-training program had been collected in a previous randomized clinical trial study [20]. Here, the raw COP data were analyzed, and were used to identify patient-specific postural control model. Details on the data, model, and the estimation of the model parameters are given below.

### Subjects, measurements and experimental protocol

Forty PD patients diagnosed based on the UK Parkinson’s Disease Society Brain Bank criteria (7 female, 63.1 ± 12.1 years; Hoehn-Yahr < 3; mini mental state examination score ≥ 24) and 20 healthy age-, height- and weight-matched control subjects (4 female, 63.8 ± 12.1 years) participated in the study. The patients were assessed before and after a 12-session balance-training program. The training program included balance exercises with different sensory stimulations and the conventional rehabilitation as well (details of clinical intervention can be found in the Appendix). The assessments of the patients were performed in the ON-medication phase, i.e. 60–90 min after taking their normal medication, consisted of clinical scales and static posturography measures. HCs were examined once and only took the posturography test. All participants provided written confirmed consent according to the Declaration of Helsinki. The Ethics committee of Iran University of Medical Sciences approved the protocol [21].

The clinical measures included Timed Up and Go (TUG) test to evaluate functional mobility as well as the Berg Balance Scale (BBS) and Functional Reach test (FRT) to assess functional balance [21].

For the posturography measures, subjects stood on a force-plate (type 9260AA6, Kistler Instrument AG, Winterthur, Switzerland) while the COP was recorded at 1 kHz for 70 s in eight trials. Stance on rigid surface with eyes open and closed (RO, RC); and standing on a 10.5 cm-thick foam with eyes open and closed (FO, FC) were performed each in two trials. The order of the abovementioned four tasks was randomized for each subject to avoid any biased result caused by learning effects. A sufficient rest interval between the trials was given to the subjects, if they needed.

### Data analysis and COP-based sway measures

COP data was filtered (10 Hz, 3rd order Butterworth) and resampled to 100 Hz. From the data (the 5–65 s of each trial), 15 common sway measures were calculated as proposed in [9] and in the anterior-posterior direction (see Additional file 1 for details of the sway measures). According to the International Society for Posture and Gait Research (ISPGR), recording duration of more than 40 s, and sampling frequency above 50 Hz guarantee steady and reliable values of the sway measures [22]. Most studies suggested 60 s of recording [23, 24], with 5 s of adjustment time before starting the recording [22, 25] to suppress the non-stationarity of the COP data, which only exists in the primary seconds of recording [23].

From all 15 measures, four representative sway measures were selected:

- *RMS*: the root mean square distance from the mean of the COP. This measure provides a measure of the sway size, and is believed to be related to the effectiveness of, or the stability achieved by the postural control system [26].
- *MV*: the mean velocity is the average of the absolute value of the COP velocity. In clinical sense, it reflects the amount of regulatory activity required to maintain stability [25]
- *f*95: the frequency associated with the 95% of the total power frequency. *f*95, besides providing an estimate of the extent of the frequency content, believed to reflect the stiffness around the ankle (the higher the *f*95 the higher the stiffness) [25].

The three above measures are widely used in the literature with high reliability and validity [10, 27]. Furthermore, these three measures can represent the three main measure groups (position-related, velocity-related, and frequency-related measures), discovered in a correlation study among all sway measures, by Maurer et al. [9].

- *∆t*_*c*_: the time coordinate for the critical point in stabilogram diffusion function (SDF) diagram [28]. *∆t*_*c*_ was also added in this study, given the strong correlation it showed with the ‘stability degree’ as will be discussed later.

These measures were used to compare patients (before training) with HCs; and to evaluate the improvement in patients after balance training. Also, the groups’ mean power spectrum density (PSD) for both COP displacement (PSD-Disp) and COP velocity (PSD-VEL) were calculated from the fast Fourier transform (see Additional file 1 for details). Although these two PSD diagrams represent COP data in the frequency-domain, they can offer a general sense for the time-domain measures. The changes in position- and velocity-related measures can be systematically interpreted considering the area under PSD-Disp and PSD-VEL, respectively. Theoretically, the area under the power spectrum of a signal accounts for the mean square value of that time series. Therefore, the area under the PSD-Disp diagram (known as *POWER*) equals the squared RMS of the COP displacement, i.e. *POWER* ≈ *RMS*^2^ [9]. In particular, the area under the frequency ranges in which the main power is concentrated is of interest (reflects an estimate of the *RMS* magnitude in PSD-Disp; and an estimate of the velocity magnitude of the COP in PSD-VEL). This proposed integrated inspection of all sway measures in the form of PSD diagrams is novel; regarding the general studies in the literature, in which the sway measures are evaluated individually [11, 29]. Finally, the COP data were used to identify postural control model parameters for each subject and task.

### Model description and parameter estimation

**Fig. 1:**
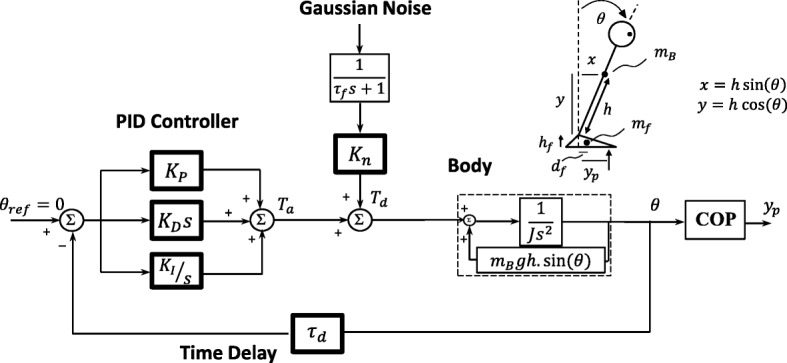
Postural control model, an inverted pendulum as ‘Body’ with PID controller representing the CNS, and time delay. The human ‘Body’ is modeled by an inverted pendulum with all mass (*m*_*B*_) centered at the height of *h*. *J* = moment of inertia of body around ankle axis; *m*_*f*_ = 2.01 kg, mass of feet; *h*_*f*_ = 0.085 m, height of the ankle axis above the ground; *d*_*f*_ = 0.052 m, the horizontal distance between the ankle axis and the center-of-mass of the feet [9]; *θ*, body sway angle, *y*_*p*_, center-of-pressure (COP) displacement. The neuromuscular controller is modeled by PID controller: *K*_*P*_ (proportional gain) main control parameter for generating corrective ankle torque; *K*_*D*_ (derivative gain), *K*_*I*_ (integral gain) control parameter responsible for undesired steady lean from upright stance. *T*_*a*_, corrective ankle torque; *T*_*d*_, disturbance torque; *K*_*n*_, internal disturbance torque gain; *τ*_*f*_ = 100 s, time constant for low-pass filter; *τ*_*d*_, time delay

The postural control model of [9] was used (Fig. 1). The model consists of an inverted pendulum, representing the biomechanics of human stance, and a PID controller (parameters *K*_*P*_, *K*_*D*_, *K*_*I*_), representing the neural control performance of the central nervous system (CNS). A disturbance torque (*T*_*d*_) in the form of a Gaussian noise was injected into the control loop to mimic the spontaneous sway – scaled by gain *K*_*n*_. The disturbance torque was filtered using a first-order low-pass filter with time constant *τ*_*f*_ = 100 s [9] to lie in the frequency range of spontaneous sway. Mass (*m*_*B*_) and height (*h*) of the pendulum were subject-specifically adjusted based on the anthropometric data of each subject [30]. The output of the model is COP displacement (*y*_*p*_). COP displacement was calculated from the body sway angle (*θ*), considering the dynamics of the inverted pendulum and feet, as formulated in Eq. 1 [9].

1 $$ {y}_p=\frac{\left({m}_B{h}^2-J\right)\ddot{\theta}+{m}_Bx\ \left(g+\kern0.5em \ddot{y}\right)-{m}_B\ddot{x}\left(y+{h}_f\right)+{m}_f{d}_fg}{\left({m}_B+{m}_f\right)g+{m}_B\ddot{y}} $$

where *x* = *h*.sin(*θ*), *y* = *h*.cos(*θ*), *g* = 9.81 m/sec^2^. *J* is the moment of inertia of the body around the ankle axis, *m*_*f*_ = 2.01 kg is the mass of feet, *h*_*f*_ = 0.085 m is the height of the ankle axis above the ground, *d*_*f*_ = 0.052 m is the horizontal distance between the ankle axis and the center-of-mass of the feet.

The PID control parameters (*K*_*P*_, *K*_*D*_, *K*_*I*_) are responsible for generating the needed corrective ankle torque (*T*_*a*_) for the stability of the ‘Body’ system. Among three PID control parameters, *K*_*P*_ (proportional gain) mainly produces this corrective ankle torque and therefore relates to the ‘stability degree’. *K*_*I*_ (integral gain) is responsible for correcting any accumulated error from upright stance, which stands for the undesired steady lean. *K*_*D*_ (derivative gain) adjusts damping around the ankle. *τ*_*d*_, time delay, corresponds to the time delay that CNS takes to respond.

From control engineering viewpoint, the balance control is defined in frequency domain. In other words, control parameters are tuned based on how adequately the power of each frequency component in the output of the system (i.e. COP displacement) is controlled in a limited bound. In this regard, the three PID control parameters shape the frequency content of the COP data. On the other side, *K*_*n*_ exclusively scale up/down the sway amplitude, irrespective of shaping the frequency content or addressing the ‘stability degree’ of any subject. For further illustration of the two different roles of the control parameters and *K*_*n*_, two sets of simulation were carried out. 1) In the first set, *K*_*P*_ was changed from *K*_*P*_ = 15.4 to 23 N.m/deg.; 2) and in the second set, *K*_*n*_ ranged from *K*_*n*_ = 300 to 600; while keeping other parameters constant (*K*_*D*_ = 5.0 N.m.sec/deg., *K*_*I*_ = 1.5 N.m/deg./sec, *τ*_*d*_ = 150 ms, *K*_*n*_ = 500 (for simulations set 1), *K*_*P*_ = 22.0 N.m/deg. (for simulations set 2)). The range of parameters were determined considering the values estimated for the HCs in task RO (as described below), as well as the extent to which the parameters ranged for PD group or other tasks.

The model parameters (*K*_*P*_, *K*_*D*_, *K*_*I*_*, K*_*n*_, *τ*_*d*_) were obtained for each subject and each task by model optimization [9]. Unlike the method of [9], results of [31] motivated us to additionally include *K*_*I*_ in our optimization algorithm. In this method, the sum of normalized differences of the 15 sway measures from the subject and the model output was chosen as the cost function (*F*_*cost*_). The minimum of *F*_*cost*_ was searched using a gradient descent algorithm by *fminsearch* MATLAB v.8.1 (Mathworks Inc., MA, USA). In order to avoid local minima, a two-level optimization technique was applied. The 5-dimensional parameter search space (with limit values of *K*_*P*_: [12, 35] N.m/deg., *K*_*D*_: [2.5,7.5] N.m.sec/deg., *K*_*I*_: [0.1,2] N.m/deg./sec, *K*_*n*_: [300,2000], *τ*_*d*_: [80,200] ms, covering the greatest extent before instability or unreasonable simulation results) was meshed (each parameter with 5 grades) to 5^5^ = 3125 grid points. First, *F*_*cost*_ was calculated for each grid point. Grid points with *F*_*cost*_ < 2, which roughly accounts for 1% of the total grid points, were opted as the initial conditions (IC) for the second and fine level of optimization, i.e. to be used as ICs for trials of *fminsearch*. The cut point of 2 for the cost function was decided based on the best optimization results of [9] with *F*_*cost*_ ~ 0.46. Finally, the best result from trials of *fminsearch* in the second level was taken as the final answer of the optimization algorithm. (see Additional file 1 for more details on the performance of this optimization algorithm).

### Statistical analysis

To compare PD patients before training (PD-Pre) to HCs, the sway measures as well as the model parameters were compared using a 2 × 2 × 2 mixed model analysis of variance (ANOVA). Mixed model ANOVA included two groups (PD and HC) as between-subject factor as well as two visual levels (eyes open (EO), eyes closed (EC)), and two surface conditions (rigid (R), foam (F)) as within-subject factors. The Tukey test was used for post hoc multiple comparisons. In order to evaluate the patients’ improvements, the paired sample *t*-test was done, comparing different clinical (TUG, and FRT) and posturography measures, and model parameters before and after training. Clinical improvement in BBS was tested with non-parametric Wilcoxon signed-rank test. The significance level was set at 0.05. Moreover, the relationship between the percent changes of sway measures and clinical improvements were calculated with Pearson correlation test.

## Results

The results are presented in three main sections: clinical measures, sway measures, and model parameters. The fourth section links the role of model parameters to changes in sway measures, with presenting model simulation results.

### Clinical outcomes

Table 1 shows the clinical measures of PD patients before and after balance training. The score of all clinical measures were improved after training, proving the effectiveness of the intervention.

**Table 1:** Clinical measures of PD patients before and after balance training

Clinical measures (unit)	Mean (Standard Deviation)		
	Before training	After training	*p*-value
Berg Balance Scale	50.8 (2.9)	53.2 (3.2)	< 0.001
Functional Reach Test (cm)	26.87 (6.86)	30.69 (7.91)	< 0.001
Timed Up and go (sec)	9.11 (4.04)	7.70 (3.51)	< 0.001

Among all sway measures, only percent changes of *∆t*_*c*_ in tasks FO and FC, showed correlation with clinical improvement in FRT (FO: *r* = − 0.419, *P* = 0.009; FC: *r* = − 0.356, *P* = 0.042).

### COP-based sway measures of subjects

**Fig. 2:**
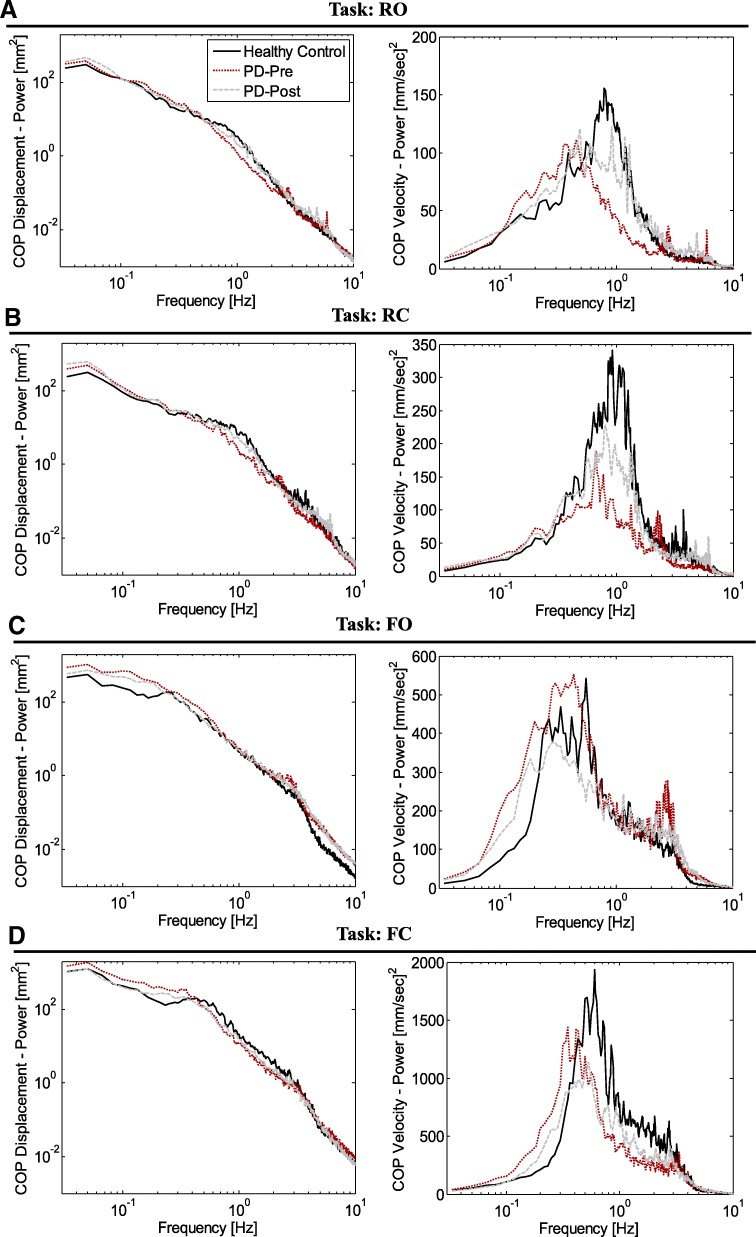
Group mean Power Spectral Density (PSD) diagrams. PSD diagrams for COP displacement (left) and COP velocity (right) for PD patients before (PD-Pre) and after (PD-Post) balance training, as well as healthy control subjects in four tasks (**a** to **d**)

Figure 2 presents the mean PSD of the COP displacement (PSD-Disp) and the mean PSD of the COP velocity (PSD-VEL), for HCs and patients in Pre and Post training, and in all four tasks (RO, RC, FO, and FC). As seen in Fig. 2, a great deal of power in the PSD-Disp is concentrated in lower frequencies (< 0.2–0.3 Hz), which corresponds to the *RMS*. Distinct differences in *RMS* (power of low frequencies) between HCs and PD-Pre, as well as PD-Pre and PD-Post were mainly in F-tasks (Fig. 2c, d). Likewise, the main power of COP velocity in PSD-VEL is expressed in the mid-range frequencies (0.2–2 Hz, this range may shift slightly in different tasks), which gives an estimate of *MV*. Distinct power differences in mid-frequencies are observed in R-tasks (Fig. 2a, b). A typical frequency shift (change in *f*95) in the bell-shaped peaks of the PSD-VELs of the three groups (HCs, PD-Pre, PD-Post) are seen mainly in F-tasks.

**Fig. 3:**
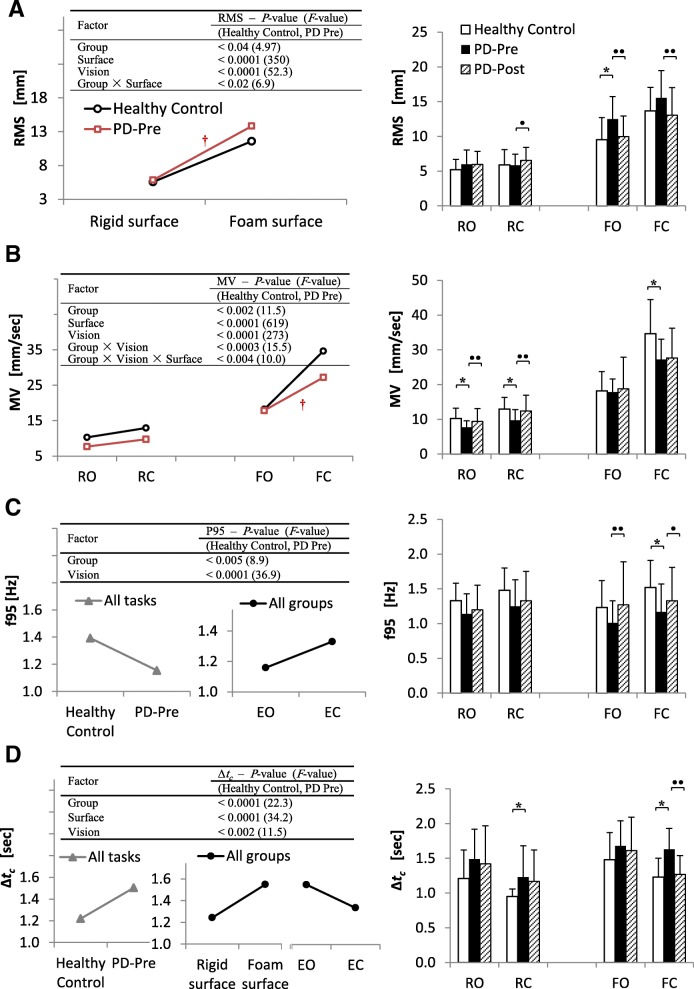
Sway measures for healthy control subjects (HCs) and PD patients before (PD-Pre) and after (PD-Post) balance training. **a** Root Mean Square (*RMS*), **b** Mean Velocity (*MV*), **c** The frequency up to which 95% of the total power frequency lies (*f*95), **d** Time coordinate for the critical point in the stabilogram diffusion function (SDF) diagram (*∆t*_*c*_). Left: ANOVA results comparing HCs and PD-Pre, †: Significant interaction (*p* < 0.05). Right: results of Tukey post hoc multiple comparisons between HCs and PD-Pre: * (*p* < 0.05). Bar charts also show paired sample *t*-test results between PD-Pre and PD-Post: • (*p* < 0.05), •• (*p* < 0.013)

Figure 3 shows the ANOVA results, comparing HCs and PD-Pre; as well as outcomes from the post hoc multiple comparisons on the four sway measures (all 15 measures are provided in Additional file 1: Table S1). Additionally, this figure presents the results of paired *t*-tests between PD-Pre and PD-Post.

#### Healthy controls vs. PD patients before training

*RMS*: Patients showed higher *RMS* (group effect: *P* = 0.03, Fig. 3a), particularly appeared in F-tasks (Fig. 3a, group × surface = 0.011, FO: *P* = 0.013). Unlike F-tasks, *RMS* was almost similar between the two groups in R-tasks.

*MV* (Fig. 3b): The ANOVA pointed out a lower velocity in PD-Pre than HCs (group effect, *P* = 0.001), with significance in R-tasks (RO: *P* = 0.005, RC: *P* = 0.0003). In addition, group by vision as well as group by vision by surface conditions significantly interacted (*P* = 0.003); particularly, patients did not increase their *MV* as much as HCs did. Unlike R-tasks, patients and HCs exhibited similar velocity in F-tasks (except for FC: *P* = 0.0003).

*f95* (Fig. 3c): Group effect was significant (*P* = 0.004), with lower *f*95 for PD-Pre (FC: *P* = 0.008).

*∆t*_*c*_ (Fig. 3d): *∆t*_*c*_ was higher for patients (group effect: *P* < 0.0001) compared with HCs (RC: *P* = 0.05, FC: *P* = 0.0004).

#### Visual- and surface-induced effects in sway measures

*RMS* goes higher on foam compared with rigid surface, and EC compared with EO (significant main effects of surface and vision). Likewise, foam surface compared with rigid surface, and EC compared with EO condition (significant surface and vision main effects) evoked faster sway, i.e. higher *MV*. As for frequency measures, *f*95 rose in EC condition (vision effect). *∆t*_*c*_ decreased with eye closure and increased on foam surface (visual effect: *P* = 0.001, and surface main effect). All except those mentioned had *P* < 0.0001, Fig. 3a-d.

#### PD patients pre and post balance training

##### R-tasks

Lower velocity (*MV*) in patients, which was mainly manifested in R-tasks, was increased by balance training (RO: *P* = 0.001, RC: *P* = 0.00006; Fig. 3b) Increase in *MV* in R-task was accompanied by a modest increase in *RMS* (RC: *P* = 0.049, Fig. 3a). No significant changes in *f*95, as well as *∆t*_*c*_, were achieved in R-tasks via training.

##### F-tasks

Balance training prompted significant reduction in *RMS* of the patients in F-tasks (FO: *P* = 0.000002, FC: *P* = 0.006, Fig. 3a). A significant shift of *f*95 to higher values is observed in two F-tasks (FO: *P* = 0.006, FC: *P* = 0.048; Fig. 3c). *∆t*_*c*_, the other frequency-related measure, although dropped in general, showed significant decrease only in FC (*P* = 0.000006, Fig. 3d). Unlike R-tasks, *MV* showed no significant improvement in F-tasks.

### Estimated model parameters

**Fig. 4:**
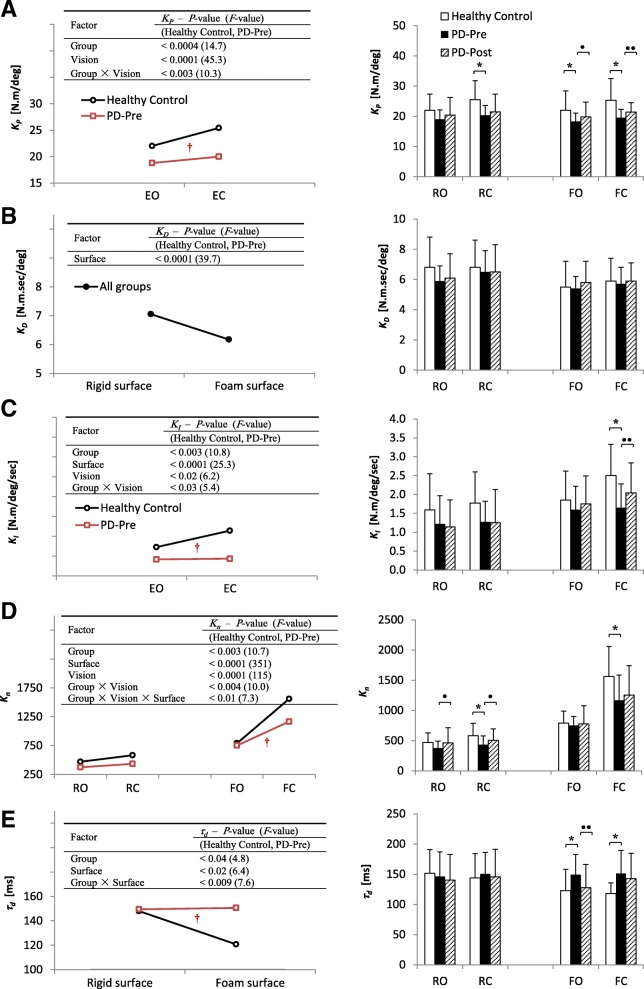
Estimated model parameters for healthy control subjects (HCs) and PD patients before (PD-Pre) and after (PD-Post) balance training. **a** *K*_*P*_ (proportional gain), **b** *K*_*D*_ (derivative gain), **c** *K*_*I*_ (integral gain), **d** *K*_*n*_ (internal disturbance torque gain), **e** *τ*_*d*_ (time delay). Left: ANOVA results comparing HCs and PD-Pre, †: Significant interaction (*p* < 0.05). Right: results of Tukey post hoc multiple comparisons between HCs and PD-Pre: * (*p* < 0.05). Bar chart also show paired sample *t*-test results between PD-Pre and PD-Post: • (*p* < 0.05), •• (*p* < 0.013)

Figure 4 shows the estimated model parameters for HCs, PD-Pre, and PD-Post. In Fig. 4, the ANOVA results as well as post hoc comparisons are shown (more details in Additional file 1: Table S2). Figure 4 also presents the results of paired *t*-tests between PD-Pre and PD-Post.

#### Healthy controls vs. PD patients before training

Patients with PD showed lower values than HCs in most of the model parameters (Fig. 4). *K*_*P*_ was significantly lower for PD-Pre compared to HCs. Nevertheless, group by vision interacted (*P* = 0.002); i.e. PD patients did not increase their *K*_*p*_ as much as HCs did in EC condition (Fig. 4a, RC: *P* = 0.0001, FO: *P* = 0.03, FC: *P* = 0.0002). Except the main effect of surface (*P* < 0.0001), all other factors were non-significant on *K*_*D*_ (Fig. 4b). PD patients performed with an abnormally low *K*_*I*_ in EC tasks (significant group × vision effect: *P* = 0.024, RC: *P* = 0.07 close to significance, FC: *P* = 0.0002, Fig. 4c).

Group significance (*P* = 0.002) emphasizes on general lower *K*_*n*_ for patients, mainly in R-tasks (RC: *P* = 0.003), and only in FC among all F-tasks (FC: *P* = 0.0004, Fig. 4d). Furthermore, similar to *MV*, *K*_*n*_ also showed group × vision as well as group × vision × surface (*P* = 0.009) interactions which recalls PD patients’ deficiency in increasing *K*_*n*_ (as well as *MV*) in task FC. As for time delay – *τ*_*d*_ –, patients displayed higher delay, particularly on F-tasks (group × surface: *P* = 0.008, FO: *P* = 0.02, FC: *P* = 0.003; Fig. 4e).

#### Visual- and surface-induced effects in model parameters

As for the significant main effects of visual and surface conditions, *K*_*P*_ adopted higher values with closing eyes. The only significant effect on *K*_*D*_ was a surface effect, which made a significant drop of *K*_*D*_ on foam. Both *K*_*I*_ (*P* = 0.015) and *K*_*n*_ rose with closing eyes and standing on foam. *τ*_*d*_ only showed significant changes for surface condition (*P* = 0.014), with a sharp drop on foam. All except those mentioned had *P* < 0.0001, Fig. 4a-e.

#### PD patients pre and post balance training

Most of the parameters for patients improved toward HC values (Fig. 4). *K*_*P*_ in patients was increased slightly in all tasks; Nonetheless, improvement in *K*_*P*_ was significant only in F-tasks (FO: *P* = 0.043, FC: *P* = 0.007). *K*_*D*_ showed no marked changes. Patients’ low *K*_*I*_ in EC conditions remarkably enhanced in FC (*P* = 0.009).

Similar to *MV*, *K*_*n*_ in patients enhanced markedly in R-tasks (RO: *P* = 0.026, RC: *P* = 0.017, Fig. 4d). Delayed response in patients (higher *τ*_*d*_) on F-tasks, was significantly decreased in FO (*P* = 0.005); while FC did not improve (Fig. 4e).

### Model simulation

**Fig. 5:**
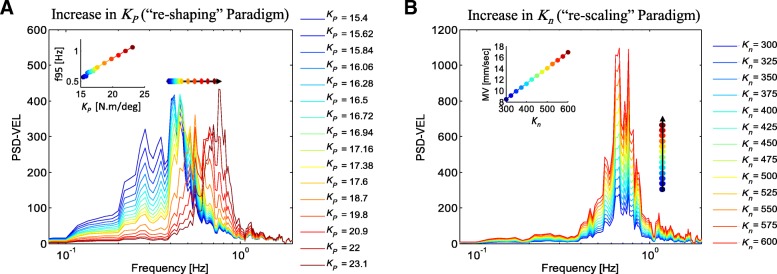
Power spectral density diagrams for COP velocity (PSD-VEL) from model simulations for different values of *K*_*P*_ and *K*_*n*_. **a** Increase in *K*_*P*_ is associated with “re-shaping” and frequency shift (change in *f*95) in the PSD-VEL. **b** Increase in *K*_*n*_ is associated with “re-scaling” in power spectral, and increase in velocity-related measures (*MV*). Parameter settings: *K*_*D*_ = 5.0 N.m.sec/deg., *K*_*I*_ = 1.5 N.m/deg./sec, *τ*_*d*_ = 150 ms, *K*_*n*_ = 500 (for simulations in **a**), *K*_*P*_ = 22.0 N.m/deg. (for simulations in **b**)

Figure 5 shows the PSD-VEL of the COP, generated from model simulations for different values of *K*_*P*_ and *K*_*n*_.

As seen in Fig. 5, increase in *K*_*P*_ is associated with frequency shift in PSD (increase in *f*95). This change pattern, in which the power of the frequency components are changed differently and hence takes a new shape will be called as “re-shaping” in the rest of this paper. On the other hand, increase in *K*_*n*_ exclusively re-scale the power of each frequency component uniformly, without contributing to the shape of the frequency content. This latter pattern will be referred to as “re-scaling” paradigm.

## Discussion

Posturography measures reflect the overall outcome of several underlying neurophysiological mechanisms. Therefore, they may fail in explaining the origin of the neurophysiological improvements [3] or may provide conflicting interpretations [1, 4], particularly when used individually [13]. To address this problem, a new evaluation framework is proposed and investigated, based on the parameters of the postural control model previously presented in the literature [9].

### PSD diagram, a tool for comprehensive study of all sway measures

The PSD diagrams for HCs, PD-Pre, and PD-Post in Fig. 2, unraveled that the differences in sway measures in these groups were originated from two main change patterns. From this perspective, the “re-scaling” paradigm appeared mainly in R-tasks; and the “re-shaping” paradigm mainly in F-tasks. Therefore, “re-scaling” caused significant differences of *MV* in R-tasks, between HCs and PD-Pre, as well as improvement in *MV* for PD-Post. In contrast, the “re-shaping” caused frequency shifts in F-tasks, which appeared as significant differences in *f*95 of the HCs and PD-Pre. Particularly, the high *RMS* in PD-Pre compared to HCs in F-tasks (Fig. 2c, d, low frequencies) arose from the “re-shaping” paradigm.

Note that the PSD diagram is merely a graphical presentation of model parameters of the postural control model. Figure 5 clearly illustrates that the two paradigms of “re-shaping” and “re-scaling”, are indeed expressing two main model parameters (*K*_*P*_ and *K*_*n*_). In other words, these two model parameters are representing two principle components of the postural control in PD (as discussed below), as well as two main recovery patterns appeared in these patients.

### Patients’ impairments and effects of balance training

#### *K*_*n*_ quantifies the ‘flexibility degree’ in patients

Patients had lower velocity in R-tasks. Velocity increased after training, which was due to patients’ improved flexibility after training. Similar behavior was observed for *K*_*n*_; suggesting that *MV* is much sensitive to *K*_*n*_ (in-line with correlation study in [9]). This correspondence points out the “re-scaling” paradigm, which occurred for patients in R-tasks after training. Hence, considering the improvement in *MV* as the expression of improved flexibility in posturography, *K*_*n*_ in the model exclusively quantified the ‘flexibility degree’ in PD. The remarked improvement of mobility in patients after training, with power increase in mid-frequency range (i.e. increased *MV*), was previously reported for elderly balance training [32] as well as in PD [33–35]. Similarly, medication and brain stimulations have attenuated axial stiffness, which to surprise of many, further increased the patients’ *RMS*, which was larger than HCs’ *RMS* at baseline [7, 11, 12].

“Re-scaling” archetype is supposed to result in escalation of power in both low-frequency (*RMS*) and mid-frequency bands (*MV*). Yet, one should be cautious about concurrent effects of *K*_*P*_ and *K*_*n*_ on *RMS* (simultaneous occurrence of re-shaping and re-scaling). Patients’ *RMS* in R-tasks before training was similar to HCs, and was barely improved after training. Lower *K*_*P*_ in patients, which also did not significantly improve after balance training in R-tasks, maintained *RMS* at low values for patients even after training.

#### *K*_*P*_ quantifies the ‘stability degree’ in postural control

Lower *f*95, higher *∆t*_*c*_, and higher *RMS* were the three sway measures with significant difference for PD-Pre vs. HCs in F-tasks. The differences in these measures were explained by lower *K*_*P*_ for patients (re-shape of PSD with shift to lower frequencies). Although higher RMS in PD-Pre on foam might stem from inadequacy of *K*_*P*_ (while *K*_*n*_ has approximately identical values), ANOVA expressed that group × surface interaction in *RMS* was in association with the same interaction in time delay among all model parameters. Indeed, patients could not adapt their time response properly with faster response needed for stability on foam. Balance training developed sufficient ankle torque production (amplifying *K*_*P*_) as well as quick response (*τ*_*d*_); both lead to reduce the *RMS*. Reduction in *RMS* on foam after training program was also observed for healthy elderly subjects [36, 37]. Moreover, reduced corrective torque due to the irregular co-contraction of muscles was numerously reported for PD [6, 18, 19, 38]. This abnormal motor set causes reduced stabilization ability reflected in lower *K*_*P*_ in our model.

As far as “re-shaping” paradigm is concerned, *K*_*P*_ has great influence on frequency content and particularly on *f*95 (Fig. 5). However, Improvement in *K*_*P*_ after training was dominantly significant in FC, the only task in which significant decrease in *∆t*_*c*_ appeared. This finding may suggest that *∆t*_*c*_ is much reliable in detection and assessment of ‘stability degree’ in PD. This is mainly because high frequency components of the COP are reflected as high resonant oscillation in stabilogram diffusion function (SDF) diagram [13]; rather than shift in time coordinate of the critical point. Furthermore, PD patients have high-frequency tremors, which considerably differ from the frequencies of the stability-band (bell-shaped peak in PSD-VEL). Therefore, *f*95 can be misleading with artifacts from tremor inputs. Moreover, only *∆t*_*c*_ among all sway measures (specifically in F-tasks) showed correlation with FRT, the clinical measure which seems to purely assess the stability. The negative relation showed that as much as *∆t*_*c*_ decreases, the FRT (i.e. the stability) increases. Raymaker et al. also recognized that *∆t*_*c*_ carry a specific information of balance, which they failed to find a meaningful expression for [39].

#### Impaired leaning perception in eyes-closed (EC) tasks in PD

EC tasks revealed a deficit in PD patients in properly increasing *K*_*I*_. By closing eyes, any individual is supposed to adopt higher *K*_*I*_, which is a measure correcting the undesired steady deviation from upright stance, i.e. undesired lean. This patients’ disability was much profound in FC, in which improvements were also achieved after training. Blaszczyk et al. also detected abnormal leaning condition in EC task for PD patients [40]. Likewise, Hue et al. observed decrease in mean COP for elderly after physical activity program and only in FC task [36].

#### Fear phenomenon in patients while standing on foam with eyes closed (task FC)

Velocity (and *K*_*n*_) on foam were similar for both groups except for FC task; implying that patients exhibited similar needed agility on foam except when they closed their eyes. Under this condition, patients displayed an unusual stiffened response with lower *MV* (and *K*_*n*_), and with similar *RMS*. This over-constraint behavior was observed before, for patients with PD in challenging tasks such as difficult cognitive tasks [41], and standing with feet in 45° configuration [42]. Interestingly, aroused fear in threatening tasks in healthy adults and patients with phobic postural vertigo caused a stiffening response too [32]. Balance training did not have any remarkable impact on this phenomenon.

### Clinical implication

#### Stability and flexibility aspects of postural control tangles together, mislead interpretation of sway measures

Manifestation of both inter-segmental rigidity and poor balance control in PD caused discrepancy in posturography results [4, 7, 11]. Hence, different training programs can bring about different or even contradictory results [35, 43]. Some interventions mainly improve ‘stability’ [44], while others might mainly improve ‘flexibility’ [35]. The new framework in the form of *K*_*P*_ and *K*_*n*_ allowed for discrimination of ‘stability’ from ‘rigidity’. This new description for stability, particularly for PD patients with upper limb tremor as one of their main symptoms, allows us to recognize stability problems from tremor-induced frequency measures. In this sense, increase or decrease in *RMS*, *MV*, or *f*95 cannot correctly address improvements; rather, the projection of these measures on the model with increment and/or drop in *K*_*P*_ and *K*_*n*_ will explain patients’ improvement.

#### Different mechanisms of balance training vs. medication

Patients with PD are usually believed to have higher *RMS*, *MV* and *f*95 [11, 12, 29]. *RMS* was increased, and *MV* and *f*95 were decreased with L-dopa therapy [1, 11, 12]. It should be strongly emphasized that this behavior is a phase change from OFF- to ON-medication states for patients; which is marked with amelioration of ‘tremor and rigidity’. Furthermore, the study by Rocchi et al. [45] indicated that MV in OFF medication correlates to frequency-related measures and specifically tremor inputs. Whereas, *MV* in ON medication is associated with sway magnitude. In other words, decrease in *MV* and *f*95 through medication is a sign of tremor reduction, rather than contributions from changes in stability (*K*_*P*_). The change of medication phase caused an increment in *∆t*_*c*_ for PD patients (0.54 s in OFF state to 1.47 in ON state) [13]. This increase in *∆t*_*c*_ was explained by decrease in *K*_*P*_ [13]. However, patients in ON-medication state still had higher ∆*t*_*c*_ compared to HCs (∆*t*_*c*_ = 1.3 s for HCs). Surprisingly, in our study, the high value of ∆*t*_*c*_ for patients in ON-medication state decreased to the value of HCs via training; which was reflected as the increase in patients’ *K*_*P*_ in our study. These reverse changes suggest a different mechanism of medication versus balance exercises. It is likely that balance training is more concerned with stability improvement, while medication is mostly effective in rigidity reduction.

### Recommendations for targeted interventions

Typical behavior of model parameters in each specific task put forth a fresh insight for the design of new targeted assessments and exercises. In this regard, EC condition induces larger *RMS* and *MV* in agreement with higher *K*_*n*_. Additionally, human seem to increase *K*_*P*_ in EC to keep themselves tighter in their base of support; a natural response from CNS for maintaining higher safety margin. This phenomenon can nicely be seen in previous PSD studies of COP [15, 32]. *K*_*I*_ also increased with eye closure, but is specifically challenged by FC condition. Consequently, exercises in EC condition may allow for enhancement of mobility, stability, and proprioceptive perception of upright stance.

Compliant surface excited higher *MV*, *RMS*, and thus *K*_*n*_. Furthermore, *K*_*D*_ was significantly lower on foam. In fact, stability on foam necessitates lower values of *K*_*D*_. The balance system needs to reduce damping to respond in an agile fashion on the compliant surface of the foam. Similarly, significant surface factor for *τ*_*d*_ showed the natural strategy CNS adopts to maintain balance on foam, i.e. to reduce response time. Therefore, exercises on foam may provide proper timing as well as mobility and agility.

### Model limitation and future work

A two-degree-of-freedom (2-DOF) double inverted pendulum model is much liable for precise demonstration of inter-segmental coupling and rigidity (body orientation). Furthermore, a 2-DOF model has the capacity of studying impaired usage of hip strategy [18, 46]. The hip strategy certainly contributes more in F-tasks. In this regard, motion capture and perturbation-based assessments can provide richer information [3, 18, 19]. In addition, our model was developed only in sagittal plane, and the mediolateral component of instability is completely disregarded here. However, many studies emphasized the emergence of postural instability in PD especially in the frontal plane [12, 40]. Some even believe in the assessment of mediolateral direction as an early detector of PD [1, 47]. Furthermore, our model lacks passive stiffness and damping of the ankle joint. Maurer et al. [9] found unsatisfactory fit of model to COP data, considering such elements. The contribution of passive elements can be a topic of future study. The poor representation of female population in our study is another limitation of this work.

Based on our PSD study and distinct implication of each frequency band, it sounds necessary for common COP-based assessments to include a new set of range-specific frequency measures instead of simple *f*50 or *f*95.

As the proof-of-concept for the proposed ‘intervention assessment tool’, future studies are needed to apply this scheme to different intervention techniques. Such studies, during a course of intervention, would give valuable information on the recovery dynamics and related model adaptations.

## Conclusion

A new framework for quantitative evaluation of postural control in patients with PD was proposed. Our results show that multiple aspects contributing to the postural instability in PD can be quantitatively disentangled by projecting posturography measures on a postural control model. Particularly, low *K*_*P*_ expresses poor ‘stability degree’, and low *K*_*n*_ indicates less ‘flexibility’ in PD. Moreover, the model can indicate specific abnormalities in patients that were not self-evident (e.g. delayed response in F-tasks, and incorrect leaning perception under EC condition). Furthermore, a novel approach for the integrated investigation of sway measures in the form of PSD diagrams was presented. PSD diagrams are a promising graphical tool for the presentation of the two ‘flexibility’ and ‘stability’ aspects in terms of “re-scaling” and “re-shaping” paradigms, respectively. Balance training helped patients to strengthen the balance control (increase *K*_*P*_), improve mobility (increase *K*_*n*_), and quickly adjust their response while standing on foam (reduce *τ*_*d*_). Hence, the framework is sensitive to improvements in ‘stability’ and ‘flexibility’ degrees of postural control in PD. As a result, different effects of each therapeutic method on postural control of PD patients can clearly be classified in light of model parameters; thereby providing future targeted assessments and interventions.

### Additional file

Additional file 1: Details on sway measures and model parameter calculations. (DOCX 237 kb)

## Data Availability

The data analyzed during the current study are available from the corresponding author on reasonable request.

## Appendix

### Clinical intervention

Patients received 12 sessions of balance exercises (4 weeks, 3 sessions per week, 45–60 min per session; with extra 30 min of conventional rehabilitation in each session) based on the task difficulty and safety of patients, in an outpatient rehabilitation center. Balance exercises included maintaining balance in different conditions (e.g., quiet standing, tandem standing, semi-tandem standing, etc.) while receiving the following six types of sensory stimulation: 1. Proprioceptive stimulation (using vibrator and different support surfaces), 2. Visual stimulation (tracking different images and videos displayed on the monitor in front of the patients), 3. Vestibular stimulation (using balance board and different movements of the head), 4. Combined proprioceptive and vestibular stimulations, 5. Combined proprioceptive and visual stimulations, and 6. Combined visual and vestibular stimulations. All patients completed the conventional rehabilitation and 12-session balance exercises and none of them reported any side effect.

## Abbreviations

BBS: Berg balance scale
COP: Center-of-pressure
EC: Eyes closed
EO: Eyes open
FC: Foam surface with eyes closed task
FO: Foam surface with eyes open task
FRT: Functional reach test
F-tasks: Foam-surface tasks
HCs: Healthy control subjects
MV: Mean velocity
PD: Parkinson’s disease
PSD: Power spectral density
PSD-Disp: Power spectral density of the COP displacement
PSD-VEL: Power spectral density of the COP velocity
RC: Rigid surface with eyes closed task
RMS: Root mean square
RO: Rigid surface with eyes open task
R-tasks: Rigid-surface tasks
SDF: Stabilogram diffusion function
TUG: Timed Up and Go test
