# Supplementary material for: Disentangling stability and flexibility degrees in Parkinson’s disease using a computational postural control model
Source: J Neuroeng Rehabil. 2019 Aug 14;16:104. doi: 10.1186/s12984-019-0574-0 (PMC6694532; doi:10.1186/s12984-019-0574-0)
Supplement: Supplementary file 1 — Details on sway measures and model parameter calculations. (DOCX 237 kb) [file 12984_2019_574_MOESM1_ESM.docx]

***Additional file 1***

**­­­­ Disentangling Stability and Flexibility Degrees in Parkinson’s Disease Using a Computational Postural Control Model**

**Zahra Rahmati, Alfred C. Schouten, Saeed Behzadipour*, Ghorban Taghizadeh, Keikhosrow Firoozbakhsh**

*** Correspondence:** Corresponding Author: [behzadipour@sharif.edu](mailto:behzadipour@sharif.edu)

1. **List of Sway Measures**

The Center-of-pressure (COP) time series is first offset-removed by subtracting its mean value: *y*(*n*) = COP(*n*) – $\bar{\mathrm{COP}}$.

| Symbol (name) |  | Formula/Description |  | Dimension |
| --- | --- | --- | --- | --- |
| **Spatial measures**  MD (Mean Distance) |  | $MD= \frac{1}{N} \sum_{n=1}^{N} \vert y\left( n \right)\vert$ |  | mm |
| RMS (Root Mean Square) |  | $RMS= \sqrt{\frac{1}{N} \sum_{n=1}^{N} {y(n)}^{2}}$ |  | mm |
| MAXD (Maximum Distance) |  | $MAXD=max\left( y \right)-\min(y)$ |  | mm |
| **Velocity measures**  MV (Mean Velocity) |  | $MV=\frac{1}{N-1}\sum_{n=1}^{N-1} \left\vert\left. \dot{y}(n) \right\vert\right.$ |  | mm/sec |
| RMSV (Root Mean Square of Velocity) |  | $RMSV=\sqrt{\frac{1}{N-1}\sum_{n=1}^{N-1} {\dot{y}(n)}^{2}}$ |  | mm/sec |
| MFREQ (Mean Frequency) |  | $MFREQ= \frac{MV}{4\sqrt{2}MD}$ |  | Hz |
| **Frequency measures^a^**  POWER (total power)  (total area under power spectrum) |  | $POWER= \mu_{0}$ |  | mm^2^ |
| f50 (50% Power frequency)  (the frequency up to which 50% of the  total power lies) |  | f50 = *f*(*u*)  where *m* is the samlles integer for which $\sum_{i=1}^{u} PSD(f\left( i \right))\geq$ 0.5*μ*_0_ |  | Hz |
| f95 (95% Power frequency)  (the frequency up to which 95% of the  total power lies) |  | f95 = *f*(*v*)  where *v* is the smallest integer for which $\sum_{i=1}^{v} PSD(f\left( i \right))\geq$ 0.95*μ*_0_ |  | Hz |

| CFREQ (Centriodal Frequency) |  | $CFREQ= \sqrt{\frac{\mu_{2}}{\mu_{0}}}$ |  | Hz |
| --- | --- | --- | --- | --- |
| FREQD (Frequency Dispersion) |  | $FREQD= \sqrt{1- \frac{{\mu_{1}}^{2}}{\mu_{0}\mu_{2}}}$ |  | — |
| **Stabilogram diffusion function (SDF) measures^b^** *D_s_* (Short-term Diffusion coefficient) |  | one-half of the slope of the regression line for the short-term region in SDF |  | mm^2^/sec |
| *D_l_* (Long-term Diffusion coefficient) |  | one-half of the slope of the regression line for the long-term region in SDF |  | mm^2^/sec |
| ∆*t_c_* |  | Time coordinate of the critical point in the SDF diagram |  | sec |
| < ∆*y_c_* > ^2^ |  | Amplitude of the critical point in the SDF diagram |  | mm^2^ |

a) Frequency measures were calculated from the Power Spectral Density (PSD) of the COP time series *y*(*n*):

*PSD*(*f*) = |*Y*(*f*)|^2^/N,

where *Y*(*f*) is the Fourier coefficients of the time series *y*(*n*). All frequency measures were calculated for the frequency range of 0.15 Hz – 10 Hz [9].

The spectral moments are defined as:

$$\mu_{k}= \sum_{i=1}^{N} {f(i)}^{k}. PSD(f\left( i \right)) , k=0,1,2$$

b) The stabilogram diffusion function (SDF) was calculated following the methods of [26]:

$${<\Delta y^{2}>}_{(m)}= \frac{1}{N-m}\sum_{i=0}^{N-m} {[y\left( i+m \right)-y\left( i \right)]}^{2}$$

Where *m* ranges from 0 to 1000, corresponding to a time shift of 0 to 10 sec between the COP traces. <·> symbolizes averaging. This analysis results in almost two distinct linear parts, known as short-term and long-term regions. One-half times the slope of the linear regression lines for each of these parts are regarded as short-term (*D_s_*) and long-term (*D_l_*) diffusion coefficients. The intersection point for the two linear fit is known as the critical point with time coordinates (∆*t_c_*) and spatial coordinate (< ∆*y_c_* > ^2^).

1. **Performance of the optimization algorithm**

Given the high-order nonlinearity of the cost function, we had to ensure that our optimization procedure finds the global minimum and did not stock in local minima. In other words, in the case of finding multiple local minima, the optimization had found different sets of model parameters, which through model simulation would result the same sway measures. In order to verify the existence of a unique solution, i.e. the global minimum, which tags exclusively a subject’s balance, a large-scale optimization was carried out for 10 representative subjects. These 10 representative large-scale optimizations included different healthy controls and patients as well as different tasks. In the large-scale optimization, the *fminsearch* was started from all grid points (totally account for 3125 Initial Conditions). The 3125 optimizations terminated to multiple solutions (one global minimum and multiple local minima). Among these multiple solutions the ones with cost function (*F_cost_* ) values lower than 0.8 (with suggestion from the best result of *F_cost_* ~ 0.46 in [9]) were chosen as the “BESTS” subset. The converged solutions of the BESTS optimizations are shown with respect to their *F_cost_* in Fig. 1.


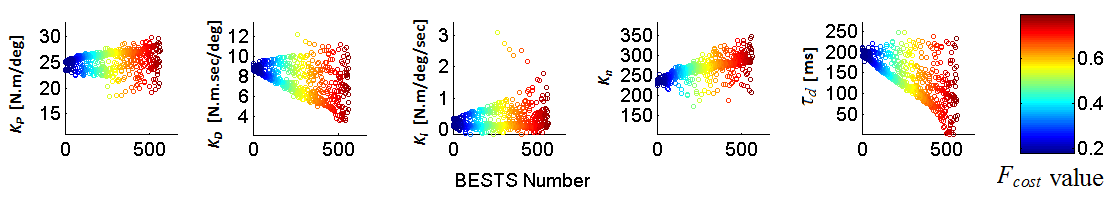


Figure 1 – Results of the large-scale optimization for the representative patient P5 in task RO: Values of the model parameters (final solutions of the optimizations) for the BESTS subset amongst all the 3125 optimizations. BESTS subset, among the whole 3125 optimization, were chosen as the optimizations with terminated *F_cost_* < 0.8.

Fig.1 shows the result for one representative subject (patient P5) in task RO. As seen in Fig. 1, all converged solutions from each distinct optimization of the BESTS subset approached a global minimum. As much as each optimization reached a lower value of *F_cost_* the terminated point was closer to the real global minimum; which guarantees the ability of the two-level optimization algorithm in finding the global minimum. This performance was observed for all 10 representative subjects and tasks. A similar performance was appeared for the sway measures, which were reproduced by simulation of the values of the model parameters of the BESTS subset (Fig. 2).


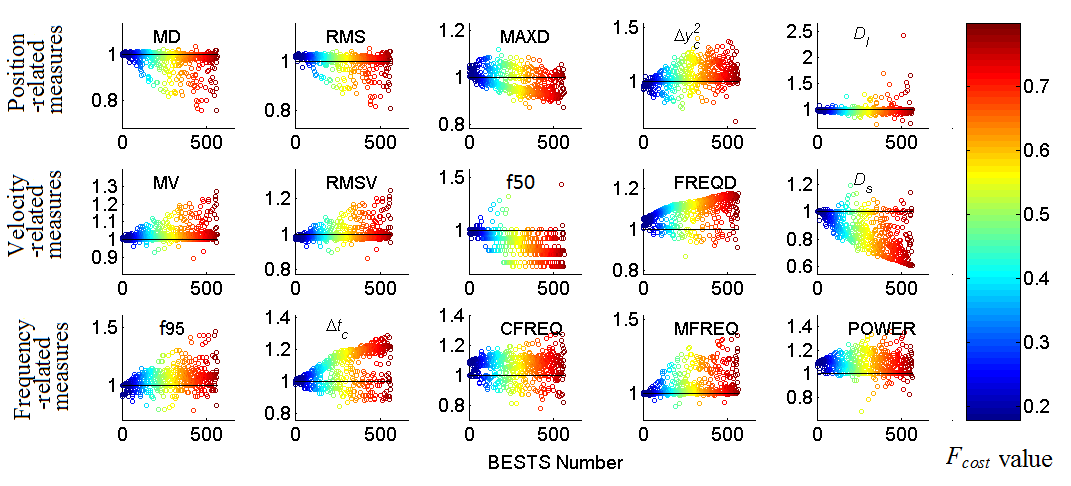


Figure 2 – Resulted sway measures from the simulations, which were set with the values of the model parameters of BESTS subset as in Fig. 1. Black line in each subplot indicates the correct value of that sway measure from the real data of the patient P5. Each measure was normalized with respect to the correct value of that measure from patient P5.

Fig. 2 shows the corresponding sway measures for the simulation result of the set of model parameters shown in Fig. 1. In addition, the black solid line in each subplot in Fig. 2 shows the correct value of each sway measure from the real data of the patient P5. Sway measures were normalized with respect to the corresponding correct value of each measure from the real data of patient P5. As seen in Fig.2, the optimization solution with the lowest *F_cost_* approaches the global solution, which correctly coincides with the correct value from the real data of patient P5.

Results promise the existence of a unique parameter explanation for each COP dataset as well as the successfulness of the utilized optimization algorithm to find this global solution.

1. **Sway measures**

Measures in table 1 are ordered based on the three principal groups found by [9] from the principle component analysis (PCA) technique: 1) position-related measures, 2) velocity-related measures and 3) frequency-related measures. Since theoretically POWER ≈ RMS^2^, POWER is not mentioned here.

Table S1 – 15 Sway measures calculated for patients with PD before and after balance training as well as healthy control subjects.

| Sway  Measures | Tasks | | | | | | | | | | | | | |
| --- | --- | --- | --- | --- | --- | --- | --- | --- | --- | --- | --- | --- | --- | --- |
|  | Healthy Control subjects (n = 20) | | | |  | PD – Pre Training (n = 40) | | | |  | PD – Post Training (n = 40) | | | |
|  | RO | RC | FO | FC |  | RO | RC | FO | FC |  | RO | RC | FO | FC |
| **Position-related measures** | | |  |  |  |  |  |  |  |  |  |  |  |  |
| MD (mm) | 4.1 ± 1.2 | 4.6 ± 1.6 | 7.5 ± 2.5 | 10.8 ± 2.7 |  | 4.7 ± 1.5 | 4.7 ± 1.4 | 9.9 ± 2.6** | 12.2 ± 3.0 |  | 5.1 ± 2.3 | 5.5 ± 2.0 | 8.1 ± 2.6‡ | 10.1 ± 2.5‡ |
| RMS (mm) | 5.2 ± 1.5 | 5.9 ± 2.2 | 9.5 ± 3.2 | 13.7 ± 3.4 |  | 5.9 ± 2.0 | 5.8 ± 1.6 | 12.5 ± 3.2** | 15.5 ± 3.9 |  | 5.9 ± 1.9 | 6.5 ± 1.9† | 10.0 ± 2.9‡ | 13.1 ± 3.9‡ |
| MAXD (mm) | 29.6 ± 8.0 | 35.4 ± 13.5 | 57.5 ± 18.4 | 82.2 ± 21.2 |  | 29.8 ± 8.3 | 31.4 ± 8.2 | 67.2 ± 14.6* | 88.8 ± 19.5 |  | 32.9 ± 11.2 | 35.7 ± 10† | 56.0 ± 15.6‡ | 73.3 ± 20.1‡ |
| < ∆*y_c_* > ^2^ (mm^2^) | 29.4 ± 16.9 | 33.3 ± 19.2 | 128.3 ± 68 | 275.3 ± 88 |  | 40.6 ± 33.4 | 39.7 ± 26.8 | 260.0 ± 146** | 435.0 ± 184** |  | 45.4 ± 42.4 | 46.4 ± 33.7 | 175.7 ± 131‡ | 278 ± 134‡ |
| *D_l_* (mm^2^/sec) | 0.96 ± 0.99 | 1.35 ± 1.33 | 1.37 ± 2.30 | 4.76 ± 4.1 |  | 1.87 ± 1.78* | 1.90 ± 1.82 | 1.31 ± 5.32 | 4.6 ± 13.6 |  | 2.37 ± 2.7 | 2.16 ± 2.11 | 1.5 ± 2.7 | 4.2 ± 5.3 |
| **Velocity-related measures** | | |  |  |  |  |  |  |  |  |  |  |  |  |
| MV (mm/sec) | 10.3 ± 2.9 | 12.9 ± 3.4 | 18.2 ± 5.5 | 34.6 ± 9.8 |  | 7.7 ± 1.9** | 9.8 ± 3.1** | 17.9 ± 3.7 | 27.2 ± 5.8** |  | 9.4 ± 3.6‡ | 12.4 ± 4.5‡ | 18.8 ± 9.1 | 27.7 ± 8.4 |
| RMSV (mm/sec) | 13.7 ± 4.0 | 16.9 ± 5.5 | 24.1 ± 7.4 | 46.0 ± 13.4 |  | 10.8 ± 3.4** | 12.7 ± 4.0** | 23.1 ± 4.5 | 36.0 ± 7.6** |  | 13.2 ± 6.1† | 15.4 ± 5.6‡ | 22.1 ± 7.3 | 36.2 ± 11.1 |
| f50 (Hz) | 0.45 ± 0.11 | 0.52 ± 0.14 | 0.34 ± 0.07 | 0.48 ± 0.08 |  | 0.35 ± 0.06** | 0.40 ± 0.10** | 0.32 ± 0.05 | 0.37 ± 0.06** |  | 0.37 ± 0.09 | 0.44 ± 0.14 | 0.32 ± 0.08 | 0.39 ± 0.06 |
| FREQD | 0.67 ± 0.07 | 0.65 ± 0.06 | 0.69 ± 0.05 | 0.64 ± 0.05 |  | 0.72 ± 0.07* | 0.70 ± 0.07** | 0.69 ± 0.05 | 0.67 ± 0.05 |  | 0.71 ± 0.08 | 0.68 ± 0.08 | 0.73 ± 0.05‡ | 0.67 ± 0.05 |
| *D_s_* (mm^2^/sec) | 21.9 ± 13.5 | 30.6 ± 17 | 60.1 ± 30 | 167.2 ± 53 |  | 16.0 ± 14.0 | 23.1 ± 19.9 | 80.7 ± 37* | 150.2 ± 41 |  | 25.0 ± 38 | 36.7 ± 42 | 64.3 ± 42‡ | 159 ± 89 |
| **Frequency-related measures** | | |  |  |  |  |  |  |  |  |  |  |  |  |
| f95 (Hz) | 1.33 ± .25 | 1.48 ± 0.32 | 1.23 ± .39 | 1.52 ± 0.39 |  | 1.14 ± 0.29* | 1.25 ± 0.38* | 1.01 ± 0.32* | 1.17 ± .40** |  | 1.20 ± .35 | 1.33 ± .42 | 1.27 ± .62‡ | 1.33 ± .48† |
| ∆*t_c_* (sec) | 1.21 ± .41 | 0.95 ± 0.11 | 1.48 ± .39 | 1.23 ± 0.27 |  | 1.49 ± 0.43* | 1.23 ± 0.45** | 1.68 ± 0.36 | 1.63 ± .30** |  | 1.42 ± .55 | 1.17 ± .45 | 1.61 ± .48 | 1.27 ± .27‡ |
| CFREQ (Hz) | 0.78 ± 0.15 | 0.85 ± 0.15 | 0.63 ± 0.15 | 0.78 ± 0.14 |  | 0.70 ± 0.16 | 0.76 ± 0.18* | 0.61 ± 0.17 | 0.64 ± 0.13** |  | 0.71 ± 0.17 | 0.78 ± 0.22 | 0.67 ± 0.21† | 0.70 ± 0.17‡ |
| MFREQ (Hz) | 0.46 ± 0.16 | 0.60 ± 0.22 | 0.46 ± 0.12 | 0.57 ± 0.11 |  | 0.36 ± 0.14* | 0.42 ± 0.14** | 0.36 ± 0.12** | 0.43 ± 0.11** |  | 0.38 ± 0.15 | 0..45 ± 0.20 | 0.44 ± 0.20‡ | 0.53 ± 0.15‡ |

Significant difference between Healthy controls and PD-Pre, independent *t*-test: * *p* < 0.05, ** *p* < 0.013

Significant difference between PD-Pre and PD-Post, paired sample *t*-test: † *p* < 0.05, ‡ *p* < 0.013

1. **Estimated model parameters**

Table S2 – Estimated model parameters for healthy control subjects and PD patients before and after balance training

| Model  Parameter | Task | | | | | | | | | | | | | |
| --- | --- | --- | --- | --- | --- | --- | --- | --- | --- | --- | --- | --- | --- | --- |
|  | Healthy Control subjects (n = 20) | | | |  | PD – Pre Training (n = 40) | | | |  | PD – Post Training (n = 40) | | | |
|  | RO | RC | FO | FC |  | RO | RC | FO | FC |  | RO | RC | FO | FC |
| *K_P_* | 22.0 ± 5.3 | 25.5 ± 6.3 | 22.0 ± 6.4 | 25.3 ± 7.2 |  | 19.0 ± 3.1* | 20.3 ± 3.3** | 18.3 ± 2.8* | 19.5 ± 2.8** |  | 20.4 ± 5.8 | 21.5 ± 5.8 | 19.8 ± 4.9† | 21.4 ± 3.1‡ |
| *K_D_* | 6.8 ± 2.0 | 6.8 ± 1.8 | 5.5 ± 1.7 | 5.9 ± 1.5 |  | 5.9 ± 1.0 | 6.5 ± 1.4 | 5.4 ± .80 | 5.7 ± 1.1 |  | 6.1 ± 1.6 | 6.5 ± 1.8 | 5.8 ± 1.4 | 5.9 ± 1.2 |
| *K_I_* | 1.59 ± .96 | 1.77 ± .83 | 1.85 ± .77 | 2.50 ± .83 |  | 1.22 ± .75 | 1.27 ± .55** | 1.60 ± .62 | 1.65 ± .63** |  | 1.14 ± .72 | 1.25 ± .88 | 1.75 ± .74 | 2.04 ± .80‡ |
| *K_n_* | 469.7 ± 156 | 582.7 ± 205 | 790.4 ± 199 | 1560 ± 498 |  | 374.6 ± 117** | 433.2 ± 147** | 751.5 ± 147 | 1166 ± 421** |  | 462.4 ± 251† | 505.9 ± 187† | 776.1 ± 299 | 1253 ± 489 |
| *τ_d_* | 151.4 ± 39 | 143.7 ± 40 | 122.7 ± 35 | 118.0 ± 17 |  | 145.9 ± 40 | 150.4 ± 35 | 149 ± 33** | 151.3 ± 38** |  | 140.3 ± 42 | 145.5 ± 45 | 127.7 ± 38‡ | 142.5 ± 42 |

Significant difference between Healthy controls and PD-Pre, independent *t*-test: * *p* < 0.05, ** *p* < 0.013

Significant difference between PD-Pre and PD-Post, paired sample *t*-test: † *p* < 0.05, ‡ *p* < 0.013
